# The Effect Failing to Perform Extracurricular Activities Has Had on School Culture and Values Education During the COVID-19 Pandemic

**DOI:** 10.3389/fpsyg.2021.778678

**Published:** 2021-10-20

**Authors:** Sümeyye Koç, Ahmet Koç

**Affiliations:** ^1^Department of Educational Administration, Faculty of Education, Near East University, Nicosia, Cyprus; ^2^Department of Religious Education, Faculty of Theology, Hitit University, Çorum, Turkey

**Keywords:** COVID-19 pandemic, distance education, school culture, values education, extracurricular activities

## Introduction

During the COVID-19 pandemic, education has continued online almost worldwide. Understanding the nature of online learning and how it reflects on practices is essential for developing an effective international perspective for continuous improvement in learning and skills (Altinay et al., 2021). In school cultures, digital technologies forces leaders to establish a vision of effective use of technology. Digital leadership is not only use of technology; it is a strategic view of school culture to engagement and achievement (Altinay, 2015).

The concept of organizational culture has had a long history and is a subject of study attracting the attention of researchers from almost all branches of science (Yilmaz, 2019). Social interaction and culture have an important place in how schools acquire a corporate identity (Altinay et al., 2019). School culture forms based on the school community members' interactions with one another and acts as a guide for how members should behave toward one another in school (Işik, 2017). The basic values a school adopts; the stories experienced within a school's history; and that school's traditions, ceremonies, and symbols constitute the basic elements of school culture.

The changes and transformations in the hierarchy of social values through globalization have led to social problems in societies (Ersozlu et al., 2018; Koç, 2020). This has caused values to become prominent for the sustainability of universal peace and the standards of human life (Bayburt and Duman, 2020). In this context, one of schools' important duties is to have students gain the values included in their programs regarding the transfer of culture and values, to prepare students for life within the framework of these values, and to positively affect their character and identity formation (Cihan, 2014). Values education in schools is done not as a separate course but within the framework of a curriculum within all courses (Aydin and Akyol-Gürler, 2018). In this context, the aim is to impart the values to be transferred to students in schools not only in class but also in extracurricular social and cultural activities (Koç and Budak, 2021).

Many activities are carried out such as field trips, competitions, scouting, book readings, seminars, conferences, camps, tournaments, and picnics within the scope of values education. The purpose of these activities in and outside of school is to create environments where students are active, to activate many different senses, to learn by doing, and to even have fun (Yildirim, 2019). Students are believed to learn more from experiences in traditional classrooms by using education-related experiences carried out in out-of-school environments that contribute to students' socialization (Priest, 1986), and that positively affect their values (Selanik-Ay and Erbasan, 2016).

This study has been conducted to examine the effect students' inability to participate in extracurricular activities due to the COVID-19 pandemic has had on how they adapt to school culture and acquire values. This study is important in terms of presenting results regarding not being able to hold the activities in schools during the COVID-19 pandemic that had previously been held for having students adapt to school culture, gain values, and socialize. In addition, the study is expected to be a guide for the adaptation process to implement for students after a 2-year hiatus due to the COVID-19 pandemic. This is because schools should guide their students in making the proper choices and should teach students the strategies to fulfill these choices in order to minimize the damage of the negative aspects the COVID-19 pandemic process has caused.

The problem of the research can be stated as, “How has students' inability to participate in extracurricular activities due to the COVID-19 pandemic affected how they adapt to school culture and to acquiring values?

## Materials and Methods

### Study Design and Study Group

This research is conducted using a qualitative research model. Qualitative research models aim to provide an environment where in-depth research can be conducted with a group that is thought to reflect the problem situation (Büyüköztürk, 2019). In this context, the study focuses on questions of how and why regarding the research problem. After obtaining permission from the Near East University Scientific Research Ethics Committee, the participants were contacted and their opinions obtained. The participants provided the data in 2021, with their consent being obtained prior to the study.

The study group consists of 43 participants. The purposive sampling method of criterion sampling has been preferred in determining the participants. Criterion sampling works with participants who show the characteristics of predetermined criteria. The main point of criterion sampling is to select participants who are rich in information (Patton, 2018). When choosing the study group, having participants who work in different schools and in different positions and who have actively taught/administered during and before the COVID-19 process have been accepted as the criteria. The participants' characteristics are as follows with the abbreviations to be used in the article indicating these characteristics also being given: Of the 42 participants, 35 are teachers (T), 4 are assistant principals (A), and 4 are principals (P). Of the participants, 30 are male (M) and 13 are female (F); 27 are undergraduates (U), 13 have a master's degree (G) and 3 have doctorates (D). In addition, the participants work in eight different types of schools from primary school to high school, and their seniority distributions range from 5 to 25 years.

### Data Collection Tool

Before creating the semi-structured interview form used in the study, opinions on the subject were received from teachers. In addition, a literature review was made in order to benefit from similar studies. The semi-structured interview form has open-ended questions and was presented to three faculty members who are experts in the field of qualitative research for their opinions. The final version of the interview form has the following six open-ended questions that were asked to the participants:

1. What activities were held in your school prior to the COVID-19 pandemic so that your students could get to know and adapt to the school culture?
2. What activities were held in your school prior to the COVID-19 pandemic so that your students could gain national values?
3. What activities were held in your school prior to the COVID-19 pandemic so that your students could gain spiritual values?
4. How has the inability of students to participate in extracurricular activities during the COVID-19 pandemic affected how they adapt to school culture?
5. How has the inability of students to participate in extracurricular activities during the COVID-19 pandemic affected how they acquire national values?
6. How has the inability of students to participate in extracurricular activities during the COVID-19 pandemic affected their ability to acquire spiritual values?

### Data Analysis

The semi-structured interview forms were obtained from the participants and evaluated using content analysis, a qualitative research analysis method. The program MAXQDA 20.1.0 has been used in the content analysis of the data. Themes and codes were created using MAXQDA, and the results have been made more understandable through the use of a word cloud and figures. The following four main strategies of credibility, reliability, transferability, and verifiability have been used as the criteria for the validity and reliability of the qualitative findings (Yildirim and Simşek, 2016). Some of the methods applied for the validity and reliability analyses in this research include ensuring the diversity of data collection, conveying the findings with as many definitions as possible, informing the reader by presenting different views on the same theme, and presenting the research area and data set to field experts.

## Results

The themes and codes were created in accordance with the answers the participants gave to the six questions on the semi-structured interview form. In the qualitative analysis, a word cloud was first created based on the participants' opinions. Next, three themes were created based on these findings. The figures present both the code frequencies and the relationships between themes and codes.

**Figure 1:**
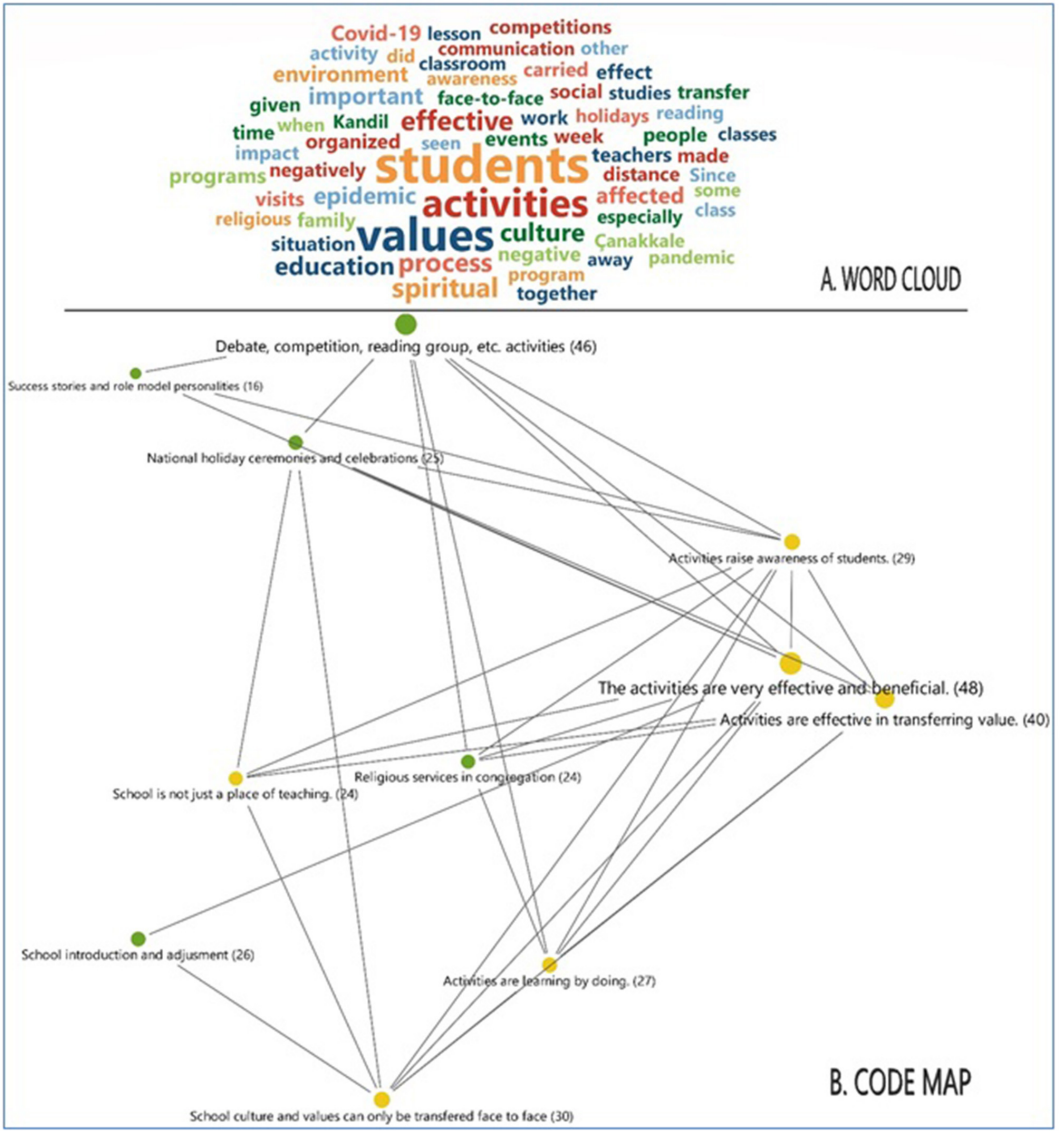
The **(A)** word cloud and **(B)** code map.

Figure 1A presents the word cloud obtained from the participants' opinions. Accordingly, the participants' extracurricular activities were determined to have an effect on school culture and values acquisition; however, the participants can be said to have the opinion that this was a negative effecte during the COVID-19 process, as the words the participants most frequently repeated were students, activities, values, effective, negatively, together, environment, pandemic, spiritual, culture, and face-to-face. Figure 1B presents the code map.

Figure 1B presents the code map describing the relationship between the effects of extracurricular activities on school culture/values acquisition and the activities implemented in schools. According to this map, the participants who feel the activities had a significant effect on the transfer of culture and values are seen to consider the activities held in their schools to have served this purpose. Almost every in-school activity has an intense relationship with culture and values transfer. For example, the participants who stated the activities to be important were determined to have significant relationship with codes from the other theme such as debate, competition, school promotion, and success stories.

### Themes and Codes

This section presents the themes and codes alongside the participants' opinions. Participants have also been coded with respect to their demographic characteristics. The definitions of these codes were explained in this article's section on the study group above. For example, TMU22 encodes teacher, male, undergraduate, and participant #22. Three themes have been created based on the participants' opinions. The first theme is the value and importance of extracurricular activities because the participants were first asked about the role activities had in conveying school culture and values. Five codes were determined for this theme by taking into account the areas in which the participants' views were most concentrated. All participants expressed the opinion that activities were very effective and beneficial. TMU22 said, “We can say that such values happen every day, maybe even every lesson without being aware of these values in the school environment. Children learn to have these values without even realizing it.” Under this theme, the participants pointed out that values, school culture can be transferred through activities, and that the school is not just a place for teaching but also a place for training. The point the participants emphasized with regard to the benefit these activities have is that these activities are a type of learning by doing, which is important for an effective education. TFG36 said, “Because peer education comes to the fore in the activities, one gains more information from one's friends and by seeing are more.” TMG1 said, “Extracurricular activities allow students to more easily internalize and reinforce values by seeing and experiencing them in the field and in daily life.” TMG28 said: “Learning by doing and through experience are most effective methods.”

The second theme is the activities implemented in culture and values acquisition. Participants who had the general view that the activities affect school culture and values acquisition were asked which activities had been held in their school. The answers they given in general terms are: competitions, debate and reading groups, national ceremonies and celebrations, collective worship, school promotion and adaptation studies, talking about the lives of role models, and conferences given by field experts. Participants stated these activities to have been carried out in their schools with effective results. TMU24 said, “Thanks to activities like trips, exhibitions, and sports activities, students can feel like they belong to the school.” PMG29 said, “Having the Gallipoli menu (only compote) being given in the school cafeteria is important so that the students can understand better what happened during the Gallipoli campaign in World War I.” TMU22 said, “We took the leftover food from our school to people in need; we had a group of students give it to them. The school also had donation boxes and sponsored orphan efforts in each classroom. Spiritual values should be given by applying them, not just theorizing about them.”

The third theme is adverse effects from the COVID-19 pandemic. While examining the activities' effects on school culture and values as the main subject of the article, educators were asked how the pandemic process had affected this since 2020. The vast majority of educators stated this process to have had negative impacts on transferring values and school culture to students, on socializing students, on students' commitment to school, and on the activities that had been previously held for these purposes. A small number of participants stated the pandemic process to have also had positive effects, such as orienting students toward spirituality and maturing them. In other words, the students said that the pandemic had caused them to think more deeply about issues such as cleanliness, death, illness, and prayer. TMU14 said, “Socialization is one of the most important elements of education; without it, the sense of belonging to a group/school does not develop.” TMG27 said, “Students couldn't benefit from the psychology of being schooled because they're always home. School definitely has an effect on people.” TMU24 said, “Children trapped at home have become unable to empathize with others.”

Among the codes created based on the participants' opinions, two codes were repeated most frequently repeated and have the most significant relationship with the other codes; these are: the COVID-19 pandemic had negatively affected the transfer of values, and COVID-19 pandemic had negatively affected the formation of school culture. Almost all participants focused on these two issues.

**Figure 2:**
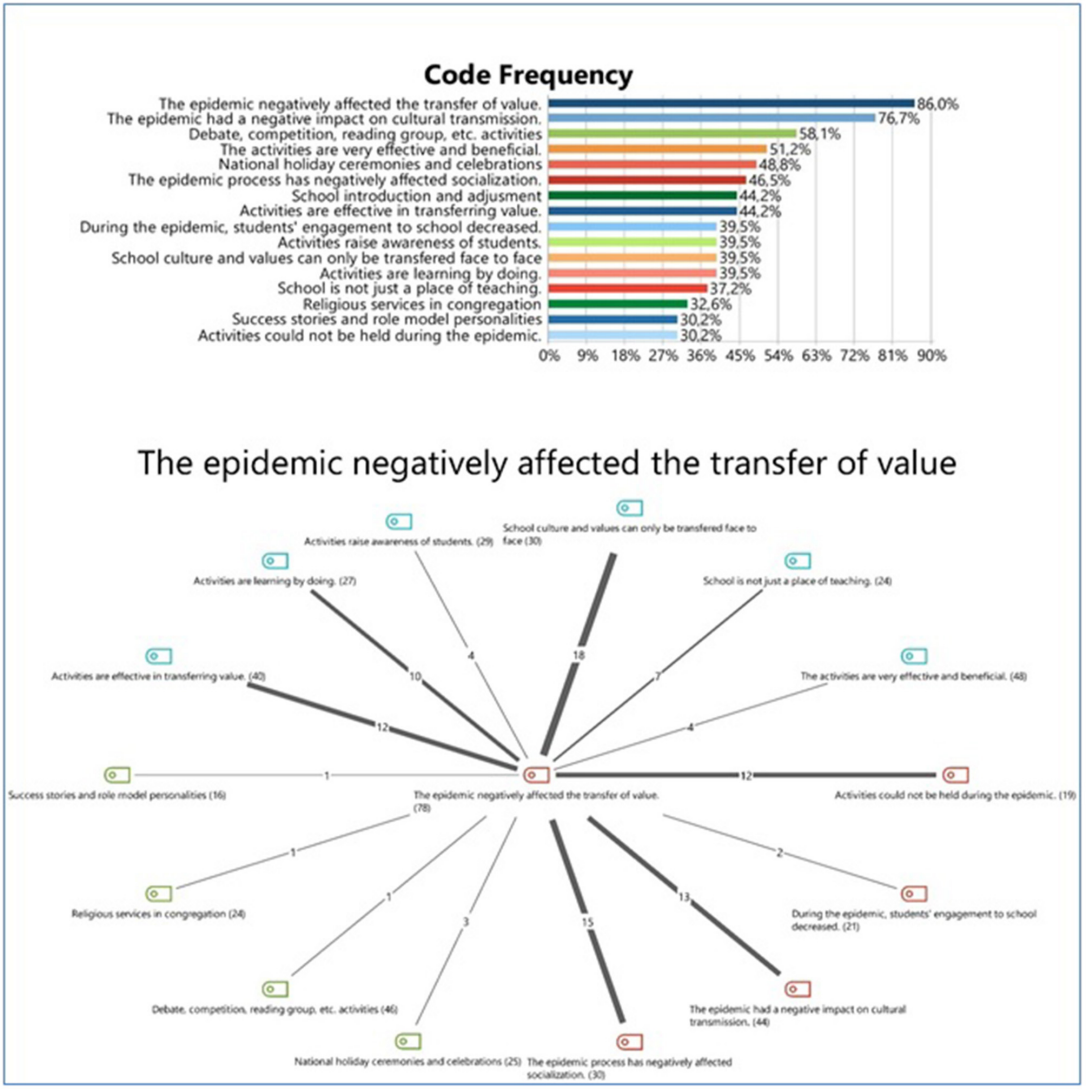
Code frequencies.

Figure 2 shows the frequencies of the codes obtained from the interviews and the relationships among the frequencies. As Figure 2 shows, the data obtained as a result of the answers participants gave reveal the following: Extracurricular activities have an indispensable importance in the transfer of school culture and values. These activities had largely been held in schools prior to the COVID-19 pandemic. However, distance education, which has been implemented during the COVID-19 pandemic, has led to social, sportive, and cultural activities being unable to be organized. As a result, students now have deficiencies adopting school culture, committing to school, knowing values, and living their lives in accordance with values. Both educators and families will need to work harder and rehabilitate children in the wake of the COVID-19 pandemic. The most striking result of this research is that extracurricular activities will play an important role in this rehabilitation work.

## Data Availability Statement

The datasets presented in this study, other related graphs and tables can be found in the online repository. The name and access address of the repository is OSF (version number 20210913): https://doi.org/10.17605/OSF.IO/WZSP4, https://osf.io/wzsp4/?view_only=dee0e6849c1d41cda7d6fd02eebdb3b0.

## Ethics Statement

The studies involving human participants were reviewed and approved by Near East University Ethical Committee Board. The patients/participants provided their written informed consent to participate in this study.

## Author Contributions

SK: conceptualization, methodology, investigation, resources, and writing/original draft preparation. AK: validation, writing/review and editing, visualization, supervision, and project administration.

## Conflict of Interest

The authors declare that the research was conducted in the absence of any commercial or financial relationships that could be construed as a potential conflict of interest.

## Publisher's Note

All claims expressed in this article are solely those of the authors and do not necessarily represent those of their affiliated organizations, or those of the publisher, the editors and the reviewers. Any product that may be evaluated in this article, or claim that may be made by its manufacturer, is not guaranteed or endorsed by the publisher.
